# Firearm Safety Screening in the Pediatric Hospital Setting: A Quality Improvement Initiative

**DOI:** 10.1097/pq9.0000000000000689

**Published:** 2023-09-28

**Authors:** Elizabeth R. Oddo, Neha Kumar, Annie L. Andrews, Stephanie Kwon

**Affiliations:** From the * Department of Pediatrics, Medical University of South Carolina, Charleston, South Carolina; † Department of Pediatrics, Baylor College of Medicine, Houston, Texas.

## Abstract

**Background:**

Firearm injuries are a leading cause of morbidity and mortality for US youth. Secure storage is protective against firearm injuries in children. Despite this evidence and national recommendations, rates of firearm safety screening among pediatric providers are low, particularly in the inpatient setting. Therefore, we aimed to increase the frequency of firearm safety screening among patients admitted to the Pediatric Hospital Medicine service.

**Methods:**

This project occurred in a tertiary pediatric hospital with a medium-sized pediatric residency program. The initial intervention was a firearm safety screening tool embedded into the electronic health record history and physical note template. Subsequent interventions included nursing education, monthly reminder emails, and gun violence discussions during intern orientation. Patients who screened positive were provided with educational materials and a free gun lock. Data collection occurred by chart review to determine the frequency of screening documentation in the H&P. A survey was also conducted among pediatric residents to identify persistent barriers to screening.

**Results:**

The percentage of inpatient firearm safety screening increased from 0.01% to 39% over 25 months, with a centerline shift noted after 2 months. Residents cited a lack of time with the patient and a belief that it was not the appropriate time to screen as persistent barriers to screening.

**Conclusions:**

This study identified an effective approach to improving firearm safety screening in an academic pediatric hospital. Hospitalization represents a unique opportunity for firearm safety screening and counseling, and inpatient providers should feel empowered to intervene in this setting.

## INTRODUCTION

### Problem Description

Firearms are the leading cause of death for youth in the United States.^1^ Thirty million US children live in a household with a firearm, and 4.6 million live in a household with at least one loaded, unlocked firearm.^2^ A study by Baxley et al showed that most children surveyed in firearm-owning households knew where their parents stored their firearms, and more than a third reported handling their parents’ firearms. Moreover, nearly a quarter of these parents did not know their children had handled the firearm at home.^3^

Simply having access to firearms poses a substantial risk to children. Nearly 90% of unintentional firearm deaths and injuries in children aged 0–14 occur in the home.^4^ In 84% of youth suicides, the firearm comes from the home.^5^ In incidents of gunfire on school grounds, 78% of shooters under 18 obtained the firearm from their home or the home of a friend or relative.^6^ Prevention of such tragic injuries is possible through secure firearm storage.

### Available Knowledge

Secure firearm storage—locked, unloaded, and separate from ammunition—is associated with decreased risk of firearm suicide and unintentional firearm injury among children. Households with locked firearms and separate locked ammunition have a 78% lower risk of self-inflected firearm injuries and an 85% lower risk of unintentional firearm injuries.^5^

The American Academy of Pediatrics recommends pediatricians routinely screen for access to firearms during patient encounters.^7^ Multiple studies have shown that physician counseling effectively produces safer storage practices.^8,9^ Most healthcare providers agree that they should provide firearm counseling, but they report many barriers, including lack of time and uncertainty of the effect.^10^ Pediatric residents, in particular, have reported a lack of training on the subject.^11^ Several studies have demonstrated low rates of firearm storage screening and safe storage counseling across multiple settings.^12,13^ In a recent study on firearm storage screening in the inpatient setting, researchers found that screening inpatient admissions for firearms in the home occurred approximately 3% of the time. In addition, only 20% of gun-owning families received anticipatory guidance on safe firearm storage.^14^

### Rationale

Previous work by Gastineau et al at our institution has shown that improving pediatric secure storage screening is possible.^15^ By implementing the “Be SMART” educational campaign in a pediatric residency primary care clinic and adding a prompt in the electronic health record (EHR) note template, the rate of firearm screening, and secure storage counseling during well-child checks increased from 3% to more than 75% in 18 months. “Be SMART” is an adult-focused firearm safety campaign to prevent firearm injuries and deaths among children through secure storage education.^16^ SMART is an acronym for Secure all guns in your home, Model responsible behavior, Ask about guns in other homes, Recognize the role of guns in suicide, and Tell your peers to be SMART. This evidence-based program increased parental knowledge of firearm safety when implemented in the inpatient setting.^9^ Given the success of Gastineau et al in the outpatient setting, we adopted a similar approach to increase firearm storage screening in the inpatient setting. We believe inpatient encounters are an important opportunity for firearm safety screening and counseling because they include many family and provider interactions, potential periods of available unstructured time compared with outpatient or emergency department visits, and a population with inconsistent primary care. Multiple other initiatives have utilized the inpatient setting for screening;^17–19^ however, with firearm injuries now being the leading cause of death for US youth, we believe screening on this subject is important now more than ever.

**Fig. 1.**
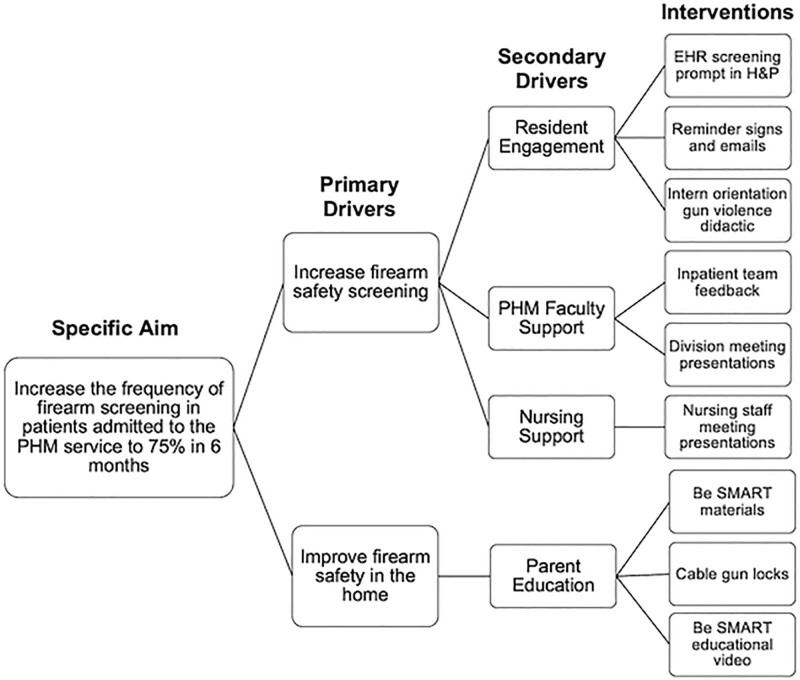
Key driver diagram reviewing the primary drivers, secondary drivers, and planned interventions for achieving our goal of increasing firearm safety screening.

The project team identified two primary drivers in accomplishing our specific aim: an implementation bundle to increase firearm storage screening and an educational package to improve firearm safety in the home (Fig. 1). We then identified four key stakeholders as secondary drivers. First, we identified residents as integral stakeholders because they would be performing the screening. Second, we considered parents so that we could provide appropriate resources if the screening was positive. Third, we enlisted support from the Pediatric Hospital Medicine (PHM) faculty to emphasize the importance of screening and counseling for the residents. Lastly, we identified nursing as important in reinforcing residents’ counseling and our institution’s firearm injury prevention culture. We used these primary drivers to develop interventions with the specific aim of increasing firearm storage screening and the global aim of improving the safety of our pediatric patients.

### Specific Aim

We aimed to increase the frequency of firearm storage screening among patients admitted to the PHM service to 75% in 6 months.

## METHODS

### Context

This QI project occurred in a tertiary pediatric hospital with a medium-sized pediatric residency program. Our institution is the only children’s hospital in the area and the largest referral center in the region, with over 5000 inpatient admissions annually. Our PHM service is composed of three inpatient teams that see a variety of common and complex diagnoses. Third- and fourth-year medical students, residents from the Pediatrics, Medicine-Pediatrics, Family Medicine, and Psychiatry programs, and PHM fellows rotate on the inpatient teams. Our institution uses Epic as our EHR (Epic Systems, Verona, Wis.).

As previously described, our pediatric residency primary care clinic successfully implemented the Be SMART educational method. In addition, a culture of gun violence prevention advocacy has been created at our institution through annual Grand Rounds presentations on pediatric firearm injuries, annual participation in “Wear Orange” National Gun Violence Awareness Day, and the recent development of a hospital-based violence intervention program (HVIP).

### Interventions

The study team included a third-year medical student, a PHM fellow, and two PHM faculty members. Table 1 depicts a timeline of the project interventions.

**Table 1.** Timeline of Interventions

Date	Intervention
November 2020	EHR prompt in H&PBe SMART materials and videoCable gun locks
January 2021	Reminder signs and emailsPHM division meetingNursing staff meeting
June 2021	Intern Community Orientation Day

## INCREASING FIREARM STORAGE SCREENING

### Resident Engagement

The interventions aimed to increase resident comfort with firearm storage screening and counseling while avoiding the impedance of inpatient workflow. The initial intervention was a firearm storage screening tool embedded into the EHR history and physical (H&P) note template as part of the social history. This tool included two yes/no screening questions: (1) Is there a gun in the home/vehicles? And (2) is the gun stored locked, unloaded, and separate from ammunition? In addition, we hung Be SMART signs in resident workrooms and placed signs reminding residents to use the screening tool on workroom computers. Also, we sent a monthly reminder email to residents starting their PHM rotation and residents scheduled to work inpatient nights. Lastly, we discussed gun violence during the new interns’ Community Orientation Day in our local area. Community Orientation Day is an annual initiative in which new interns are introduced to the local community, and the patient population they will serve throughout their residency. This day includes didactic sessions and a city tour led by a local community leader. Gun violence is discussed in an hour-long didactic session led by a pediatric hospitalist (and research team member) and members of our HVIP. Group leaders also discuss gun violence throughout the city tour, which travels directly to locations where gun violence has occurred.

### PHM Faculty Support

To enlist the support of PHM faculty members, we presented the project at bi-monthly division meetings and larger General Pediatrics monthly research meetings. We regularly updated PHM faculty members and their inpatient teams on project progress.

### Nursing Support

We notified nurses of the project through presentations made at monthly nursing staff meetings. We also instructed nursing on playing the Be SMART educational video in patients’ rooms and made them aware of available gun locks.

## IMPROVING FIREARM SAFETY IN THE HOME

### Parent Education

Residents were instructed to provide safe storage counseling using the Be SMART method if a patient screened positive for an unsecured firearm in the home. Resources available to families included Be SMART handouts and an educational video that residents could order through the EHR to play on the television screen of a patient’s room. In addition, we provided cable gun locks to interested families free of charge.

### Measures

We obtained the baseline rate of firearm screening through a manual chart review of 30 random charts per month from January to June 2020. After implementing our interventions, we collected data by weekly chart review of patients admitted to the PHM service. We excluded patients transferred from intensive care units and the newborn nursery. Our primary outcome of interest was documentation of firearm storage screening in the initial H&P. We then averaged weekly screening rates to determine monthly screening rates. Secondary outcome measures included whether a gun was in the home and if it was stored securely. Upon completion of the study period, we conducted a monthly post hoc chart review of 30 random charts from January to December 2022 to assess for long-term sustainability. We also surveyed pediatric residents to identify persistent barriers to screening.

### Analysis

We constructed a statistical process control chart and used the Institute for Healthcare Improvement rules for assessing special cause variation and trend changes. Once the data fit the criteria for a significant trend, we re-analyzed data to create new mean and control limits. We constructed process control charts using Excel. Survey data were collected and analyzed using REDCap electronic data capture tools. REDCap (Research Electronic Data Capture) is a secure, web-based software platform to support data capture for research studies.^20^

## RESULTS

**Fig. 2.**
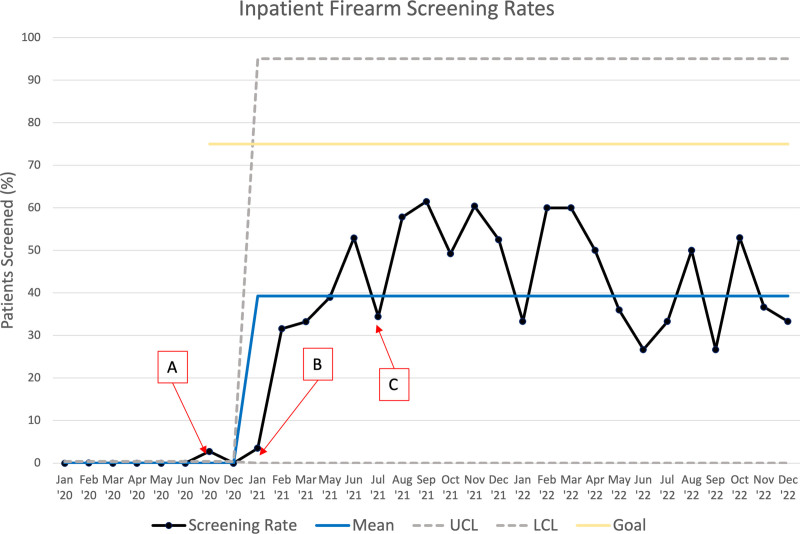
Process control chart depicting firearm safety screening rates among Pediatric Hospital Medicine Service patients. A, EHR prompt: Be SMART materials and video, cable gun lock distribution. B, Reminders: the project was presented at the Pediatric Hospital Medicine division and nursing staff meetings. C, Intern Community Orientation Day.

Data collection occurred for a total of 25 months (Fig. 2). We reviewed 180 charts in the baseline period, 847 during the study period, and 360 in the post hoc analysis. A pre-intervention chart review from January to June 2020 reveals that the baseline rate of firearm storage screening before our interventions was approximately 0.01%. Of note, the initial intervention of the EHR prompt did not result in any substantial change in screening rates. With subsequent interventions, screening rates increased, leading to a centerline shift to 39%. These interventions included implementing monthly reminder emails, signs on the workroom computers, and nursing education. The increased screening rate above the initial baseline was maintained over the following 23 months, including after the arrival of a new intern class and in the 12 months after the study period ended.

There were no instances of incomplete screening, meaning responses to both screening questions were documented in all cases. Regarding secondary measures, 27% of screened patients reported having a gun in the home or vehicle. Of these, 16% reported having a gun not stored securely.

**Fig. 3.**
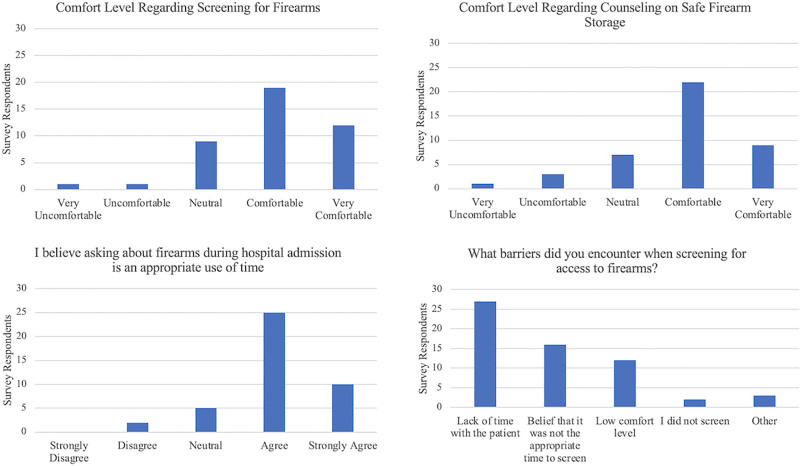
Follow-up survey responses among pediatric and medicine-pediatric residents.

We sent the follow-up survey to all 65 Pediatrics and Medicine-Pediatrics residents. We collected 42 responses for a response rate of 65%. Approximately 78% of residents stated they felt “comfortable” or “very comfortable” screening for firearms, and 74% stated they felt “comfortable” or “very comfortable” providing firearm safety counseling. In both instances, PGY-1 residents reported lower comfort levels than PGY-2-4 residents. In addition, 83% of residents agreed or strongly agreed that asking about firearms during hospital admission was an appropriate use of time. When asked about persistent barriers to screening, the most common barrier was a lack of time with the patient (64.3%), followed by the belief that it was not the appropriate time to screen (38.1%; Fig. 3).

## DISCUSSION

In summary, over the 25 months of this project, we significantly improved firearm storage screening rates among patients admitted to the PHM service. We attribute this improvement to implementing multiple interventions, including an EHR prompt, monthly reminder emails, and support from key stakeholders, particularly residents and nursing staff. Ultimately, we believe this increase in firearm storage screening will improve safe storage practices in the home, creating safer households for our patients.

### Interpretation

Interestingly, implementing the EHR prompt alone was insufficient to substantially change firearm storage screening rates. This finding contrasts with previous results obtained at our institution by Gastineau et al, in which implementing an EHR prompt in the well-child check note template significantly increased firearm storage screening from 3% to 84%. This prompt remains in use in our pediatric residency primary care clinic. We expected residents’ familiarity with the screening questions to work in our favor and translate appropriately to the inpatient setting. Of note, answering the prompt is mandatory (meaning the note cannot be signed without responding) in the clinic note template but not in the H&P note template. Making the prompt mandatory in the H&P may be an important strategy to increase inpatient screening rates.^21^ Additionally, the low screening rates may be due to the nature of the patient encounter. Anticipatory guidance is a regular part of well-child checks. Consequently, residents may feel more comfortable, including firearm storage screening during this portion of their visit. In the inpatient setting, it may seem that similarly appropriate timing does not exist during the initial history and physical. Our survey also reflects this possibility, in which 38.1% of residents believed it was not the appropriate time to screen as a persistent barrier.

Despite this belief, hospitalization can be a unique opportunity for firearm safety screening and counseling. Compared with outpatient or emergency department visits, a longer hospitalization can provide additional opportunities for family and provider interactions. Families may also have periods of unstructured time in which screening and counseling can occur. In addition, this patient population, particularly those with special healthcare needs, may have had inconsistent primary care and thus would not have received anticipatory guidance elsewhere.^22^ Lastly, as many families have limited their interactions with the healthcare system during the COVID-19 pandemic, healthcare providers should take the opportunity to screen at any encounter, whether inpatient or otherwise.

The significant centerline shift observed in our project occurred after the implementation of resident-focused reminders and the involvement of the nursing staff. This observation demonstrates the importance of the multidisciplinary team in the inpatient setting and the involvement of key stakeholders in the improvement process. We hypothesize that nursing involvement may have led to increased discussions surrounding gun violence prevention on our unit, which may have encouraged residents to screen more consistently. The significant change was maintained after two new intern classes arrived in July 2021 and 2022. During the intern orientation process and the remainder of the academic year, our institution’s culture of gun violence prevention is repeatedly reinforced through education and activism. This reinforcement helps ensure our efforts will be sustainable and continue impacting our community.

We found that 27% of screened patients reported having a gun in the home or vehicle. This finding contrasts with results from a recent study by the RAND Corporation in which the average gun ownership rate per household in our state was estimated to be 43%.^23^ Also, our rate of unsecured firearm storage (16%) was substantially lower than current national estimates (36%).^2^ These differences may be attributed to social desirability bias and parents’ hesitancy about disclosing firearms in their homes to a provider. Future studies are needed to assess ways to improve parental comfort by discussing firearm safety so that appropriate counseling and intervention can occur.

Our survey identified a lack of time with the patient as a persistent barrier to screening. This barrier is similar to the results of previous studies in which time is cited as a limiting factor to firearm safety screening.^12^ Future interventions should improve workflow and use other opportunities during the hospital stay, such as during the nursing intake or after morning rounds. Another potential solution may be to use video-based education on secure storage instead of direct provider counseling. In addition, further studies are needed to determine the ultimate impact of these interventions on safe storage behaviors in the home and the subsequent risk of firearm injury in this population. Per our institution’s trauma registry database, we, unfortunately, saw an increase in pediatric firearm injuries during our study period, indicating that our interventions may not have achieved the desired downstream effect of decreasing firearm injuries in our patient population. Finally, future directions for our project include quantifying the frequency of counseling and the amount of firearm safety resources provided to families, including cable gun lock distribution.

### Limitations

This project was not without limitations. First, interventions were implemented on a rolling basis and thus did not strictly follow structured plan-do-study-act cycles. Our team did consider balancing measures in the study design (such as additional time required to perform screening). However, these metrics proved difficult to quantify and were not included in the analysis. Although we identified parents and nursing as important stakeholders in this process, we did not include them on the study team or directly assess their beliefs on firearm safety screening. Additionally, our screening questionnaire did not directly address the number of firearms a caregiver owned and whether all were stored securely. Finally, because we could not directly observe provider–patient interactions, we used documentation in the EHR as a proxy for screening.

## CONCLUSIONS

This study identified an effective approach to improving firearm storage screening in an academic pediatric hospital. A screening tool embedded in the H&P note template, resident and nursing education, and the availability of firearm safety resources were integral to the success of this project. Hospitalization represents a unique opportunity for firearm safety screening and counseling, and inpatient providers should feel empowered to intervene in this setting.

## DISCLOSURE

The authors have no financial interest to declare in relation to the content of this article.
